# Activated Peripheral CD8 + T Lymphocytes Inhibit the Proliferation of Hippocampal Neural Stem Cells via the IFN‐γ/JAK/STAT Signaling Pathway

**DOI:** 10.1002/iid3.70287

**Published:** 2025-10-28

**Authors:** Xiaowei Li, Xiaobin Sun, Shiyu Hao, Guicheng Wang, Yanjing Guo, Yingxue He, Qidi Zhang, Zunsai Feng, Gongming Wang, Chengxiao Liu

**Affiliations:** ^1^ Department of Anesthesiology Shandong Provincial Hospital Affiliated to Shandong First Medical University Jinan China; ^2^ Department of Geriatrics the Affiliated Tai'an City Central Hospital of Qingdao University Tai'an China

**Keywords:** CD8 + T lymphocytes, IFN‐γ, JAK/STAT, neural stem cells, proliferation

## Abstract

**Background:**

The inhibition of hippocampal neurogenesis is associated with cognitive impairment in a variety of diseases, which are often accompanied by neuroinflammation. Our preliminary results revealed that the number of CD8 + T lymphocytes infiltrating the hippocampus of mice that underwent major abdominal surgery increased significantly and contributed to the inhibition of hippocampal neurogenesis and cognitive impairment. However, which stage of hippocampal neurogenesis is affected and the specific mechanisms involved remain unclear.

**Methods:**

We isolated hippocampal neural stem cells (NSCs) and peripheral CD8 + T lymphocytes from C57BL/6 mice and then used a transwell coculture system to mimic the state in which peripheral CD8 + T lymphocytes infiltrated the parenchyma but did not directly contact NSCs.

**Results:**

The results showed that activated peripheral CD8 + T lymphocytes inhibited the proliferation of NSCs in a time‐ and dose‐dependent manner. The inhibitory effect of CD8 + T lymphocytes on NSC proliferation may be achieved through the secretion of cytokines, especially IFN‐γ, as administration of IFN‐γ neutralizing antibodies could attenuate this effect. Adding IFN‐γ directly to the coculture system also inhibited NSC proliferation, even without activating T cells. Additionally, the JAK2/STAT1 pathway in NSCs was activated by activated peripheral CD8 + T lymphocytes or exogenous IFN‐γ, and a JAK2‐specific inhibitor reversed the inhibitory effect of activated peripheral CD8 + T lymphocytes on NSC proliferation.

**Conclusion:**

These results demonstrated that activated peripheral CD8 + T lymphocytes can indirectly inhibit the proliferation of hippocampal NSCs by secreting IFN‐γ and subsequently activating the JAK/STAT signaling pathway in NSCs. Our study may help to better elucidate the regulatory role of neuroinflammation in hippocampal neurogenesis, especially in some pathological states.

## Graphical abstract

Activated peripheral CD8 + T lymphocytes inhibit NSC proliferation by secreting IFN‐γ. IFN‐γ activates the JAK/STAT signaling pathway in NSCs, thereby inhibiting NSC proliferation.

## 1 Introduction

Research over the last few decades has established that neurogenesis occurs throughout life in the brains of adult rodents and primates [1]. There are two main regions of the mature brain where neural stem cells (NSCs) aggregate: the subventricular zone (SVZ) of the limbic ventricle of the lateral ventricle and the subgranular zone (SGZ) of the dentate gyrus of the hippocampus. Newborn neurons are continuously generated from the pool of NSCs and then integrated into the neural network. Adult hippocampal neurogenesis is the most robust form of plasticity in the adult brain and has been confirmed to be involved in emotion, social behavior, cognitive function, stress and other physiological functions [2]. More and more studies have shown that impaired neurogenesis in the SGZ is associated with cognitive impairment in a variety of diseases, such as depression and Alzheimer's disease [3, 4, 5]. Our previous studies also confirmed that the inhibition of hippocampal neurogenesis plays important roles in postoperative cognitive dysfunction (POCD) [6, 7].

Hippocampal neurogenesis encompasses multiple processes, including transient amplification of NSCs, giving rise to neuroblasts, differentiating into immature neurons and further maturation of fine structures [8]. The SGZ, which is a thin band between the hilus and the granule cell layer, provides a unique microenvironment for this process. Multiple intrinsic regulators coordinate with the microenvironment through various signaling pathways to regulate NSC maintenance, self‐renewal and multipotent differentiation [9]. Neuroinflammation consequent to intracranial infection, stroke, neurodegenerative disease, aging, acute and chronic stress and so forth, seems to be a crucial regulatory factor for hippocampal neurogenesis [10]. Under these pathological conditions, T lymphocytes, which are normally trapped outside the brain parenchyma, enter the brain parenchyma. T lymphocytes infiltrating the hippocampus can affect the composition and architecture of NSCs in the SGZ and impair cognitive function [11]. Our preliminary results revealed that the number of CD8 + T lymphocytes infiltrating the hippocampus of mice that underwent major abdominal surgery increased significantly and contributed to the inhibition of hippocampal neurogenesis and cognitive impairment [6]. However, which stage of hippocampal neurogenesis is affected and the specific mechanism involved remains unclear.

As important adaptive immune cells, T lymphocytes secrete a variety of cytokines to regulate the immune response. IFN‐γ, the only member of the type II interferon family, is secreted mainly by activated T lymphocytes and natural killer cells and is a pleiotropic cytokine that is expressed in a variety of neurodegenerative and neuroinflammatory disorders [12]. IFN‐γ has been reported to play important roles in regulating the proliferation of adult NSCs and neuronal differentiation in the SVZ [13]. In vivo experiments have also indicated that IFN‐γ can reduce hippocampal neurogenesis under certain pathological conditions [14, 15]. The binding of IFN‐γ to IFNGR results in the formation of a heterotetramer on the receptor, which leads to the activation of the JAK‐STAT signaling pathway. Subsequently, STAT undergoes homo/heterodimerization, translocates to the nucleus, binds to the IFN‐γ activation sequence (GAS) and the interferon‐stimulated response element (ISRE), and regulates the expression of related genes [16]. However, the effect of CD8 + T lymphocytes on the JAK/STAT pathway in NSCs in the SGZ is still unknown.

In this study, we isolated primary NSCs and peripheral CD8 + T lymphocytes from C57BL/6 mice and then used a transwell coculture system to mimic the state in which peripheral CD8 + T lymphocytes infiltrated the parenchyma but did not directly contact NSCs. We mainly focused on the effects of activated CD8 + T lymphocytes on the proliferative ability of NSCs and further explored the specific signaling mechanisms by administering exogenous IFN‐γ, IFN‐γ neutralizing antibodies and specific inhibitor of the JAK‐STAT signaling pathway. We hypothesized that activated CD8 + T cells indirectly inhibit hippocampal NSC proliferation via IFN‐γ‐mediated JAK/STAT activation.

## 2 Materials and Methods

### 2.1 Animals

Pregnant mice and male mice (C57BL/6 background) for this study were purchased from Beijing Vital River Laboratory Animal Technology Company. The mice were kept in the Laboratory Animal Center of the University, which is an enclosed SPF facility. Five to six male mice were housed per cage, and the female and male mice were housed together at a 3:1 ratio. The facility had a regular light/dark cycle, and the animals had free access to water and food. All experiments were in accordance with the National Institutes of Health Guide for the Care and Use of Laboratory Animals and ARRIVE guidelines.

### 2.2 Isolation and Culture of NSCs

Mice within 24 h of birth were used to extract primary NSCs in our experiments. They were euthanized by cervical dislocation, and their brains were rapidly removed. The hippocampus was subsequently isolated under a microscope. After the meninges were removed, single‐cell suspensions were obtained via mechanical separation. The cells were washed, briefly centrifuged, and resuspended in serum‐free DMEM/F12 (Invitrogen, USA) supplemented with 20 ng/mL basic fibroblast growth factor, 20 ng/mL epidermal growth factor (PeproTech, USA), B‐27 (without vitamin A) (Invitrogen, USA), and penicillin/streptomycin (Invitrogen, USA). The medium was changed every 3–4 days, and the cells were passaged every 7 days. All the experiments were performed on single‐cell suspensions after the second or third passage. Immunofluorescence staining and flow cytometry were used to verify the purity of the isolated NSCs.

### 2.3 Isolation of CD8 + T Lymphocytes

Whole blood was collected in sodium heparin vacutainer tubes via retro‐orbital vein puncture. Peripheral blood lymphocytes were separated with a Mouse Peripheral Lymphocytes Isolation Liquid Kit (Solarbio, China). The extract was subsequently subjected to a Mouse CD8 + T‐Cell Enrichment Kit (Thermo Fisher, USA) for further purification and enrichment of peripheral CD8 + T lymphocytes. Flow cytometry was used to verify the purity of the isolated CD8 + T lymphocytes.

### 2.4 Transwell Coculture Experiments and Activation of CD8 + T Lymphocytes

After 24 h of culture, the enriched CD8 + T lymphocytes were cocultured with NSCs in 24‐well plates. The NSCs were cultured in the large chamber of transwell 24‐well plates, the CD8 + T lymphocytes were cultured in the small chamber, and half of the medium from the NSCs was replaced with lymphocyte‐conditioned culture medium. The number of NSCs (2 × 10^5^ cells per well) was kept constant, and different numbers of CD8 + T lymphocytes were added to each well to form groupings of different proportions. All the experiments were performed using passage 1 (P1)‐P3 cultures of NSCs, which were determined to have similar multiplication rates. T cells were cocultured with NSCs for 72 h. T cells were activated with anti‐CD3 (5 µg/mL) and anti‐CD28 (5 µg/mL) (BioXcell, West Lebanon, NH) monoclonal antibodies, and the effects of these activated cells and unstimulated T cells on the proliferation of the NSCs were assessed. IFN‐γ (20 pg/mL, Servicebio, China) or AG490 (a JAK inhibitor, 10 µmol/L, MCE, USA) was added into the culture medium if necessary.

### 2.5 Ethynyl‐20‐deoxyuridine (EdU) Analysis

The NSCs and CD8 + T cells were cultured in Transwell 24‐well plates at the above concentrations, and EdU (RiboBio, CHN) (10 µM) was added to the culture medium for 72 h. The medium was discarded, and the cells were washed twice with PBS. The plates were incubated in 4% paraformaldehyde for 30 min at room temperature, incubated with glycine (2 mg/mL) in a decolorizing shaker for 5 min, and then washed three times with PBS. The cells were incubated with PBS containing 0.5% Triton X‐100 for 10 min at room temperature and then washed three times with PBS. EdU staining reaction mixture was added, and the cells were incubated away from light for 30 min at room temperature. The solution was discarded. The cells were washed three times with PBS and then stained with DAPI reaction solution away from light for 5 min at room temperature. The solution was discarded, and an anti‐fluorescence quencher was added. After all the steps were completed according to the manufacturer's instructions, the cells were observed and photographed using a fluorescence microscope (OLYMPUS BX53M).

### 2.6 Immunofluorescence

The cells were dissociated and spread on glass coverslips coated with polylysine (100 µg/mL, Solabio, China). After a 24‐h attachment period, the cells were fixed with 4% paraformaldehyde for 15–30 min at 37°C. Then, the cells were blocked with 10% donkey serum at 37°C for 1 h, incubated with primary antibodies (mouse anti‐nestin, 1:500, Abcam, UK; rabbit anti‐IFNGR1, 1:200, Abcam, UK) at 4°C overnight, and incubated with secondary antibodies (goat anti‐rabbit IgG, 1:1000, Abcam, UK; goat anti‐mouse IgG, 1:1000, Abcam, UK) at room temperature for 1 h. DAPI (10 µg/mL; C0065; Solarbio, China) was used to stain the nuclei. The sections were immediately observed under microscope (OLYMPUS BX53M) and photographed.

### 2.7 Flow Cytometry

NSCs or CD8 + T cells were fixed in 1× Fix/Perm Buffer working solution for 40 min at 4°C and washed with 1× Perm/Wash Buffer, and 80 ~ 100 µL of 1× Perm/Wash Buffer was added, after which the cells were incubated with a APC‐coupled anti‐Nestin antibody (Biolegend, USA) or FITC‐coupled anti‐CD8a antibody (Biolegend, USA) for 2 h at 4°C. Isotype‐matched antibodies were used as controls. The cells were washed with 1× Perm/Wash Buffer and incubated for 2 h at 4°C. The cells were centrifuged and resuspended in 200 ~ 300 µL of B‐PBS in each tube and analyzed within 30 min.

### 2.8 Enzyme‐Linked Immunosorbent Assay (ELISA)

After 72 h of coculture, activated or unactivated NSCs and lymphocyte coculture media were collected separately, the cell‐free supernatant was collected after centrifugation at 500*g* for 5 min, and the IFN‐γ concentration was determined using a mouse IFN‐γ enzyme‐linked immunosorbent assay kit (R&D Systems, USA) according to the manufacturer's instructions.

### 2.9 CCK‐8 Assay

NSCs and lymphocytes were cultured in 24‐well transwell plates in the coculture model described above. After 3 days of coculture, 30 µL of CCK‐8 solution was added to each well in accordance with the instructions for the CCK‐8 reagents (G4103, Servicebio, China), and then the cells were incubated for 2–4 h in the cell culture incubator. The OD was subsequently measured at 450 nm with an enzyme labeler. The relative cell viability was calculated from the OD values. All CCK‐8 assays were repeated three times.

### 2.10 Western Blot Analysis

The cells were lysed using RIPA buffer (Beyotime, China) containing 1 mmol/L PMSF to obtain proteins for electrophoresis. Proteins separated from the whole‐cell lysates were transferred to PVDF membranes (Whatman, USA). The membranes were blocked in TBS‐T for 1 h and then incubated with primary antibodies against p‐JAK1 (1:1000, CST), p‐JAK2 (1:1000, CST), t‐STAT1 (1:1000, CST), p‐STAT1‐ser (1:1000, CST), p‐STAT1‐tyr (1:1000, CST), t‐STAT3 (1:1000, CST), p‐STAT3‐ser (1:1000, CST), p‐STAT3‐tyr (1:2000, CST), and β‐actin (1:1000, Proteintech) in TBS‐T containing 5% skim milk overnight at 4°C. The membranes were then incubated with a secondary antibody (goat anti‐rabbit IgG–HRP, 1:5000, Proteintech) for 2 h at room temperature. Protein expression signals were visualized via enhanced chemiluminescence reagents (Millipore Corporation, USA).

### 2.11 Statistical Analysis

All measurement data are expressed as the mean ± standard error of the mean (SEM) from at least three independent experiments. For the EdU assays, three randomly selected fields of view under 200× magnification were counted by a third person and the average values were taken for statistical analysis. *T*‐test was performed to compare values between two groups, and one‐way ANOVA was used to compare values among multiple groups. Tukey's post hoc test was used for pairwise comparisons after one‐way ANOVA. Data analysis and image plotting were performed using GraphPad Prism 9.0 software. *p* < 0.05 was considered to indicate statistical significance.

## 3 Results

### 3.1 Isolation, Culture and Characterization of NSCs and CD8 + T Lymphocytes

**Figure 1:**
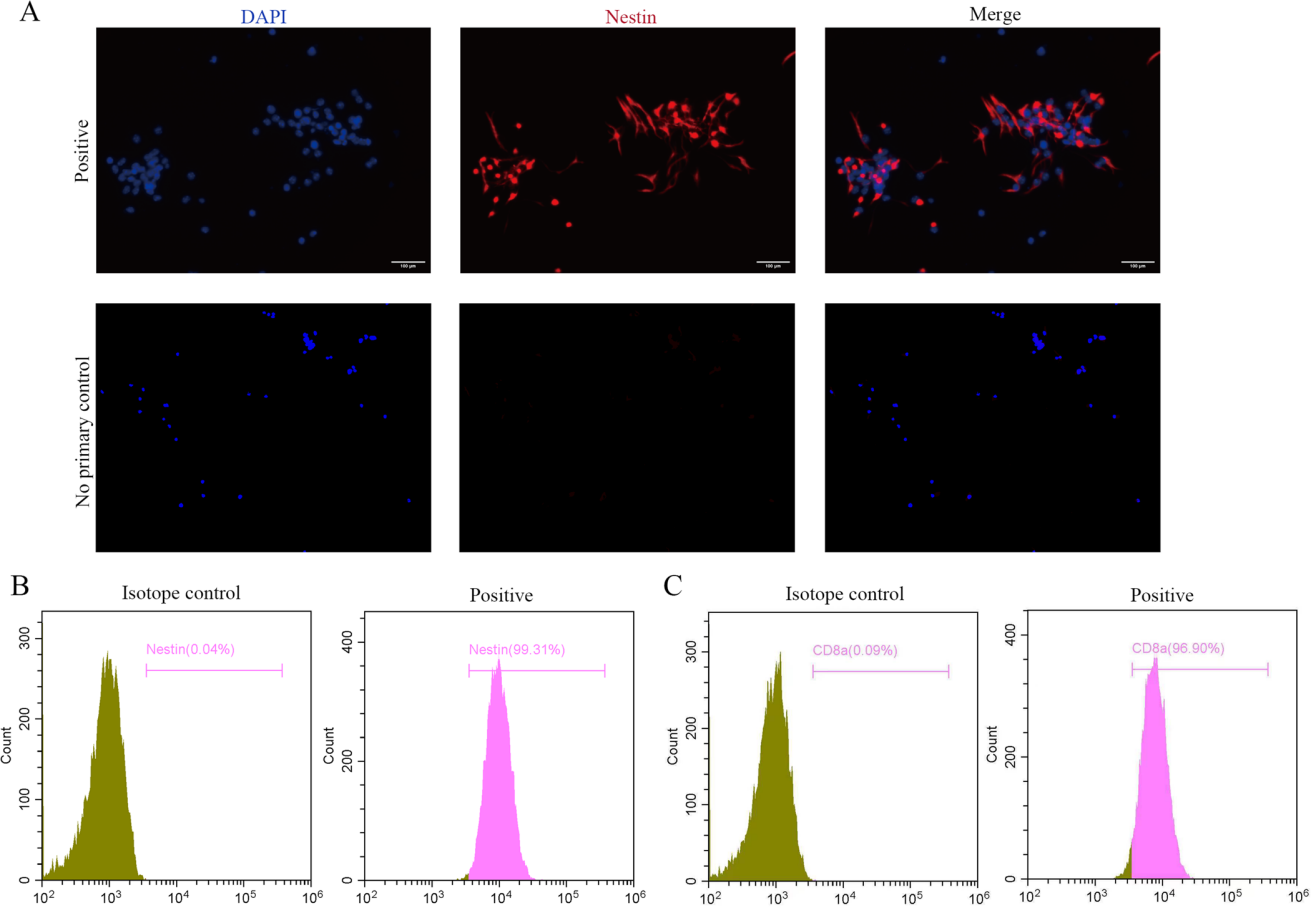
Isolation culture and identification of NSCs and CD8 + T cells. (A) Immunofluorescence image of NSCs, Nestin in red and DAPI in blue, scale bar = 100 μm; (B) Flow cytometry showed that more than 95% of the isolated and extracted cells were Nestin positive; (C) Flow cytometry showed that more than 95% of the isolated and extracted peripheral T lymphocytes were CD8 + T lymphocytes.

Primary NSCs were isolated and extracted from the hippocampus of mice 24 h after birth as described above, and the positive expression of nestin, a marker of NSCs, was confirmed by immunofluorescence staining and flow cytometry (Figure 1A,B). Flow cytometry revealed that more than 95% of the cells were positive for nestin (Figure 1B). We subsequently extracted CD8 + T lymphocytes from the blood of adult male mice. Flow cytometry revealed that more than 95% of the isolated cells were CD8 + T lymphocytes (Figure 1C). These results demonstrated that primary NSCs and peripheral CD8 + T lymphocytes were successfully isolated.

### 3.2 Inhibition of NSC Proliferation by Activated CD8 + T Lymphocytes

**Figure 2:**
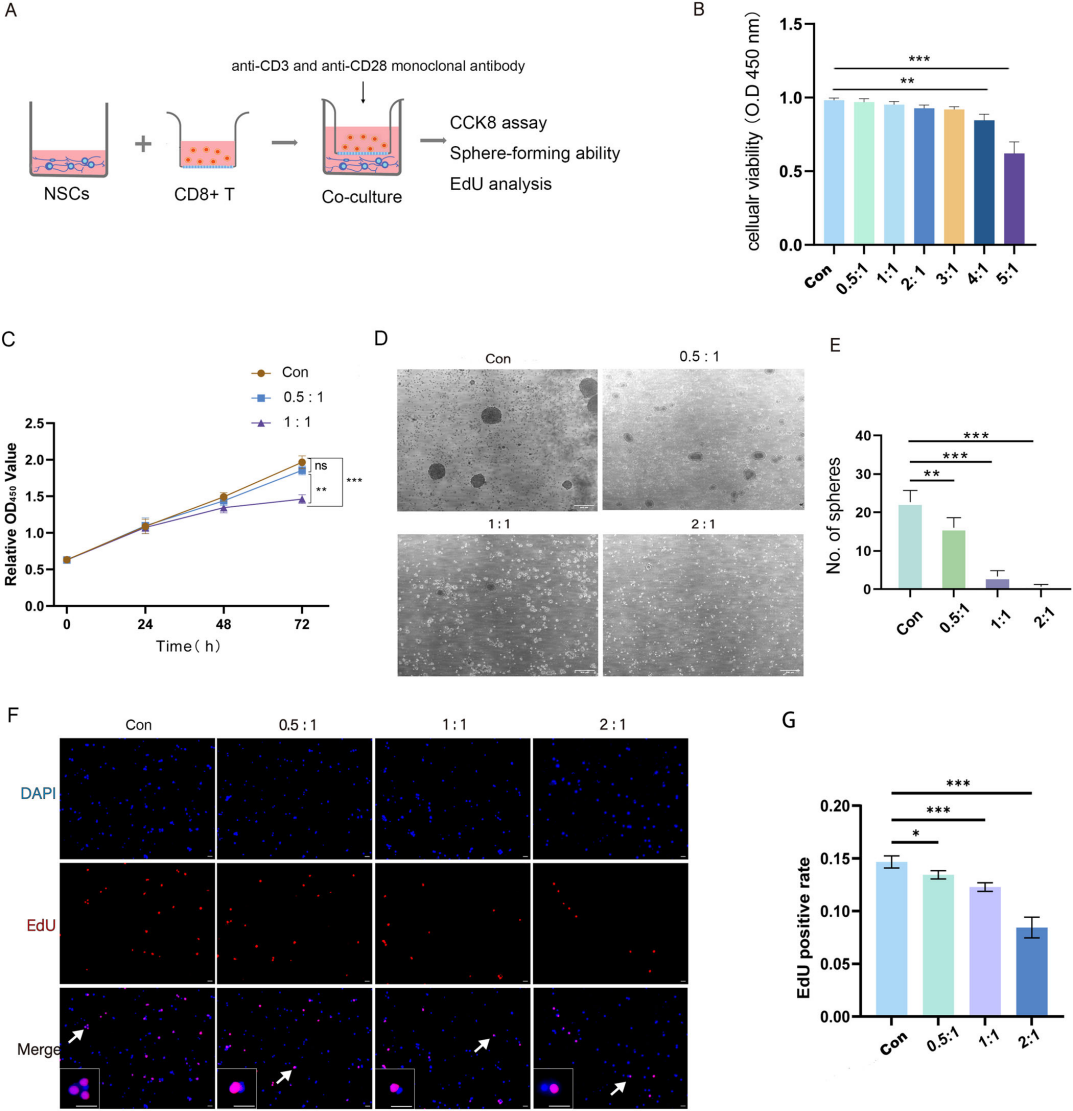
Activated T cells inhibit the proliferation of NSCs. (A) Diagram of the experimental design; (B) CCK8 assay to determine the toxic effect of activated CD8 + T cells on NSCs at different coculture ratios (*n* = 3); (C) CCK8 assay to determine the proliferation curve of NSCs after coculture at different coculture ratios (*n* = 3); (D) Light microscopy of the sphere‐forming ability of NSCs, scale bar = 500 μm; (E) Quantification of the number of neurospheres (*n* = 3); (F) Representative immunofluorescence images of EdU+ NSCs at different coculture ratios with EdU in red and DAPI in blue. The image at the bottom left showed a magnification of the point of the arrow. Scale bar = 100 μm; (G) Statistical diagram of EdU+ NSCs at different coculture ratios (*n* = 5). Unactivated, CD8 + T cells not stimulated with CD3 and CD28; n:1 = The numerical ratio of activated CD8 + T cells to NSCs was n:1. *p < 0.05, **p < 0.01, ***p < 0.001.

To mimic the state in which peripheral CD8 + T lymphocytes infiltrate the parenchyma but do not directly contact NSCs, we cocultured isolated NSCs and peripheral CD8 + T lymphocytes by using a transwell coculture system (Figure 2A). Cell viability was assessed via the CCK8 assay. The cells were grouped according to the different ratios of activated CD8 + T lymphocytes to NSCs, and we found that a high ratio of CD8 + T lymphocytes to NSCs (4:1 vs. con, *p* = 0.0075; 5:1 vs. con, *p* < 0.001) had a noticeable cytotoxic effect on NSCs 24 h after coculture (Figure 2B). The proliferation curves were generated at a low ratio of activated CD8 + T lymphocytes to NSCs, and the results revealed that a relatively low ratio of CD8 + T lymphocytes to NSCs (1:1 vs. Con, *p* < 0.001) still had a noticeable cytotoxic effect on NSCs 72 h after coculture (Figure 2C). The sphere‐forming ability of the NSCs was assessed after 72 h of coculture, and the results revealed that this ability was disrupted by coculture with activated CD8 + T lymphocytes (Figure 2D,E). An EdU incorporation assay was further performed to assess how different ratios of CD8 + T lymphocytes to NSCs affect NSC proliferation after 72 h of coculture. The results revealed NSC proliferation was more strongly inhibited as the proportion of CD8 + T cells increased (Figure 2F,G). In addition, we have added experimental data on how CD8 + T cells (at a ratio of T lymphocytes to NSCs of 0.5:1) affect NSC proliferation after different coculture durations. The results revealed that the proliferation of NSCs was noticeably inhibited after 72 h of coculture (Figure S1). In subsequent studies, a ratio of T lymphocytes to NSCs of 0.5:1 was chosen, as this ratio was closer to what has been observed in some neurodegenerative diseases, including our previous POCD model [6].

### 3.3 Activated T Lymphocytes Inhibit NSC Proliferation by Releasing IFN‐γ

**Figure 3:**
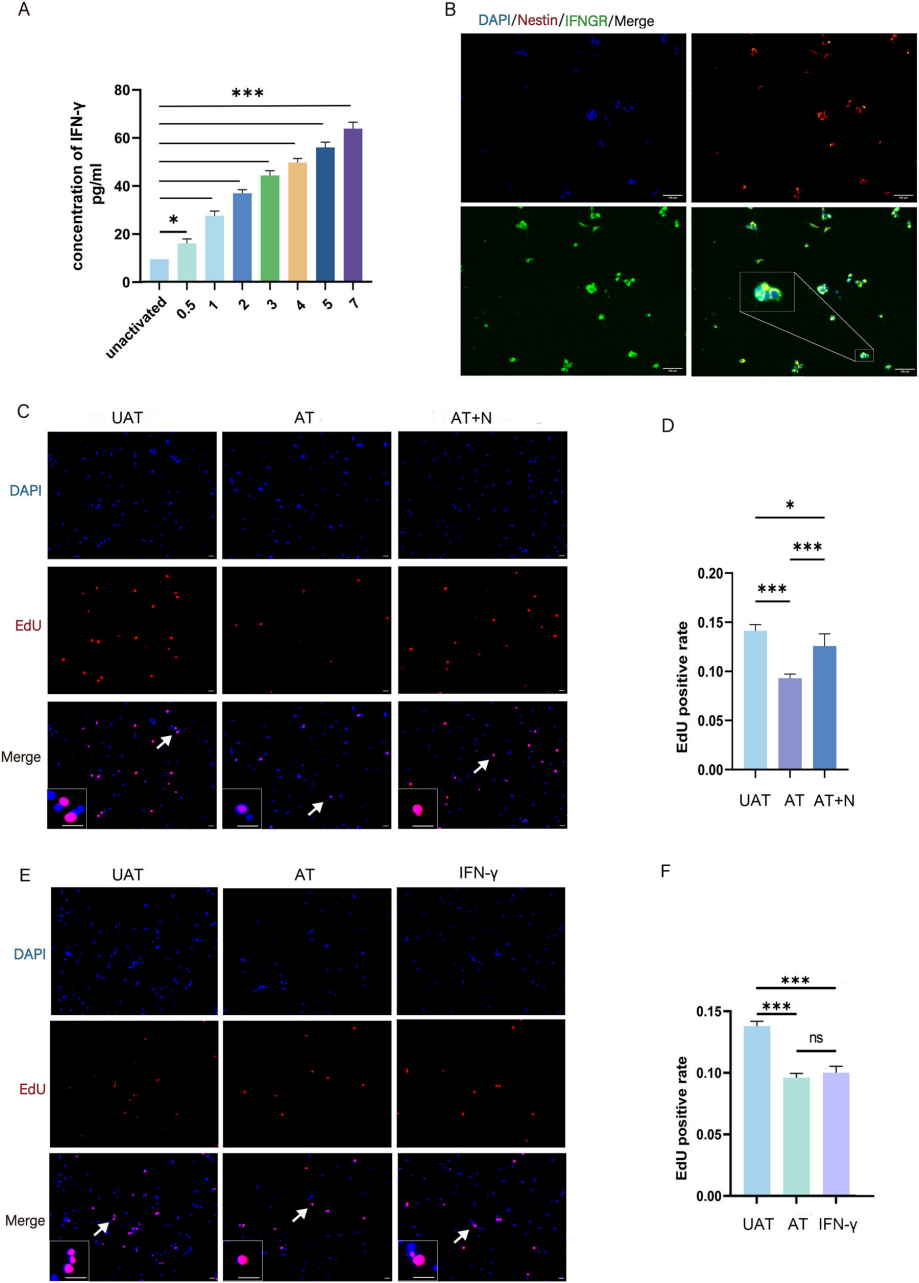
Activated T cells inhibit NSC proliferation by releasing IFN‐γ. (A) IFN‐γ secretion concentration at different scales (*n* = 3); (B) Immunofluorescence showing the presence of IFNGR on NSCs under different treatment conditions, scale bar = 100 μm; (C and E) Representative immunofluorescence images of EdU+ NSCs under different treatment conditions. The image at the bottom left showed a magnification of the point of the arrow. Scale bar = 100 μm; (D and F) Statistical diagram of EdU+ NSCs under different treatment conditions (*n* = 5). UAT, unactivated CD8 + T lymphocytes; AT, activated CD8 + T lymphocytes; AT + N, activated CD8 + T lymphocytes and IFN‐γ neutralizing antibodies. *p < 0.05, **p < 0.01, ***p < 0.001.

Having established that activated CD8 + T lymphocytes inhibit NSC proliferation, we next explored whether soluble factors produced by activated CD8 + T lymphocytes are critical for inhibiting NSC proliferation. As one of the major effector functions of CD8 + T lymphocytes is the secretion of IFN‐γ, the level of IFN‐γ in the culture supernatant was subsequently measured by capture ELISA. The concentration of IFN‐γ increased significantly over time (Figure 3A). On the other hand, our results showed that IFNGR was highly expressed on the surface of NSCs (Figure 3B).

To verify whether activated CD8 + T lymphocytes inhibited NSC proliferation through the secretion of IFN‐γ, we added IFN‐γ‐neutralizing antibodies into the coculture medium, and the results showed that the inhibitory effect of activated CD8 + T lymphocytes on NSC proliferation was significantly attenuated (Activated CD8 + T lymphocytes vs. Activated CD8 + T lymphocytes and IFN‐γ neutralizing antibodies, *p* = 0.0348) (Figure 3C,D). It was worth noting that the incomplete recovery of the proliferative ability of NSC suggested that CD8 + T cells may also inhibit NSC proliferation through other mechanisms. As perforin/granzyme B is one of the possible mechanisms, we measured the granzyme B concentration in the medium after activation of T cells and the results showed a significant increase in concentration after 72 h (Figure S2). In addition, adding IFN‐γ directly into the coculture medium inhibited the proliferation of NSCs even if CD8 + T lymphocytes were not activated (Unactivated CD8 + T lymphocytes vs. IFN‐γ, *p* < 0.001) (Figures 3E,F).

### 3.4 IFN‐γ Phosphorylates JAK/STAT Pathway Components in NSCs

The conventional signaling pathway activated by IFN‐γ involves sequential phosphorylation of tyrosine residues on JAK and STAT proteins, which is the main mechanism by which this pathway regulates gene expression [17]. As previous studies have shown that JAK1/JAK2 and STAT1/STAT3 are involved mainly in IFN‐γ signaling, we analyzed the levels of phosphorylation of JAK1/JAK2 and STAT1/STAT3 at different sites in cocultured NSCs by Western blot analysis.

**Figure 4:**
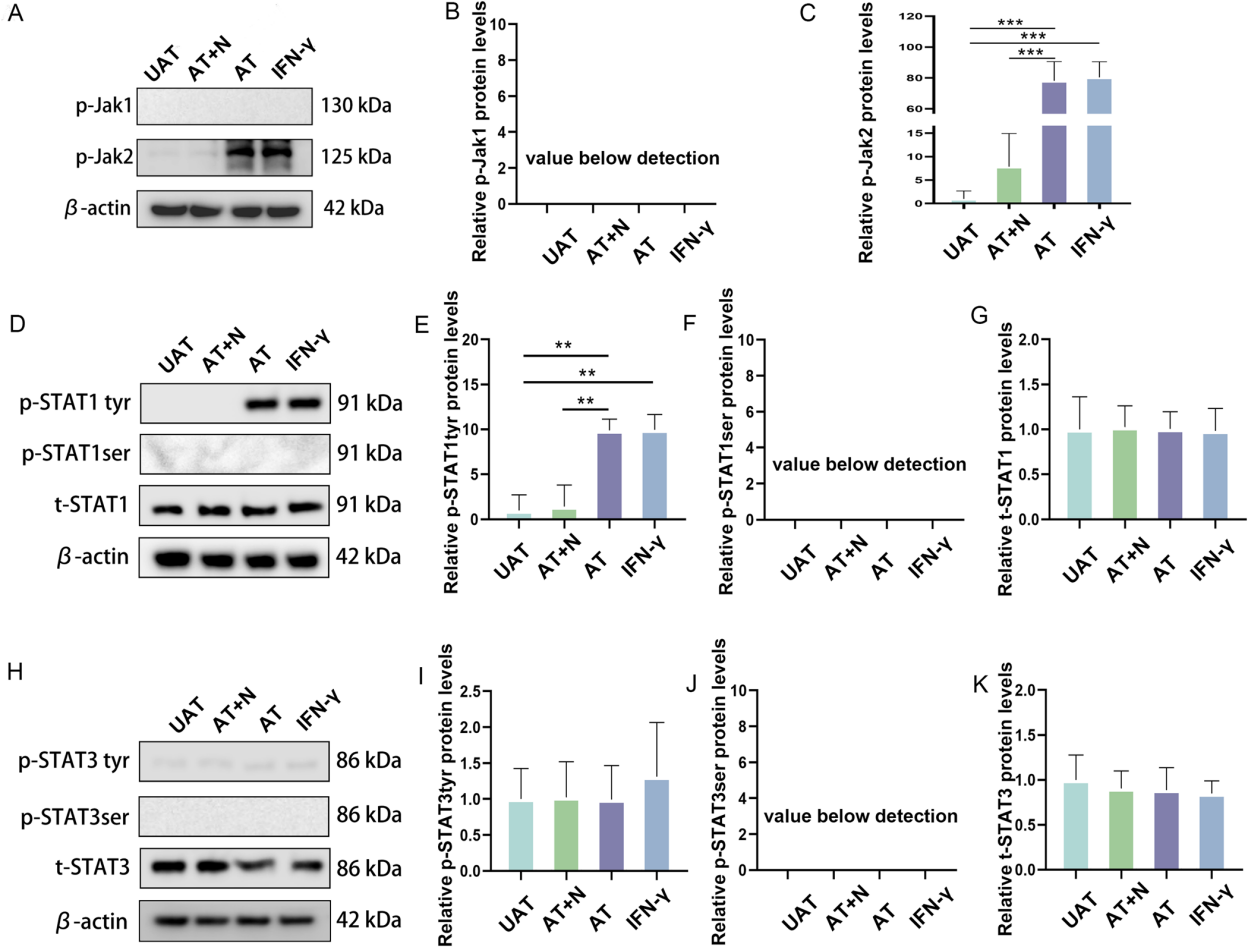
IFN‐γ secreted by activated CD8 + T lymphocytes causes the phosphorylation of JAK2 and STAT1 in NSCs. (A) Representative western blot image of p‐Jak1 and p‐Jak2 expression in NSCs under different treatment conditions; (B and C) Relative expression of p‐Jak1 and p‐Jak2 proteins; (D) Representative western blot image of p‐STAT1‐tyr, p‐STAT1‐ser and t‐STAT1 expression in NSCs under different treatment conditions; (E–G) Relative expression of p‐STAT1‐tyr, p‐STAT1‐ser and t‐STAT1 proteins; (H) Representative western blot image of p‐STAT3‐tyr, p‐STAT3‐ser and t‐STAT3 expression in NSCs under different treatment conditions; (I–K) Relative expression of p‐STAT1‐tyr, p‐STAT1‐ser and t‐STAT1 proteins. AT, activated CD8 + T lymphocytes; AT + N, activated CD8 + T lymphocytes and IFN‐γ neutralizing antibodies; UAT, unactivated CD8 + T lymphocytes. *N* = 3/group. *p < 0.05, **p < 0.01, ***p < 0.001.

In NSCs cocultured with activated CD8 + T lymphocytes or exogenous IFN‐γ, JAK2 was activated via phosphorylation (UTA vs. AT, *p* < 0.001; UTA vs. IFN‐γ, *p* < 0.001), which was reversed by IFN‐γ neutralizing antibodies (AT + N vs. AT, *p* < 0.001) (Figure 4A–C). Similar expression levels of phosphorylated STAT1 (Tyr701) were also observed (UTA vs. AT, *p* = 0.001; UTA vs. IFN‐γ, *p* = 0.001; AT + N vs. AT, *p* = 0.002) (Figure 4D–K).

### 3.5 Phosphorylation of JAK/STAT Pathway Components Mediates the Inhibitory Effect of Activated CD8 + T Lymphocytes on NSC Proliferation

**Figure 5:**
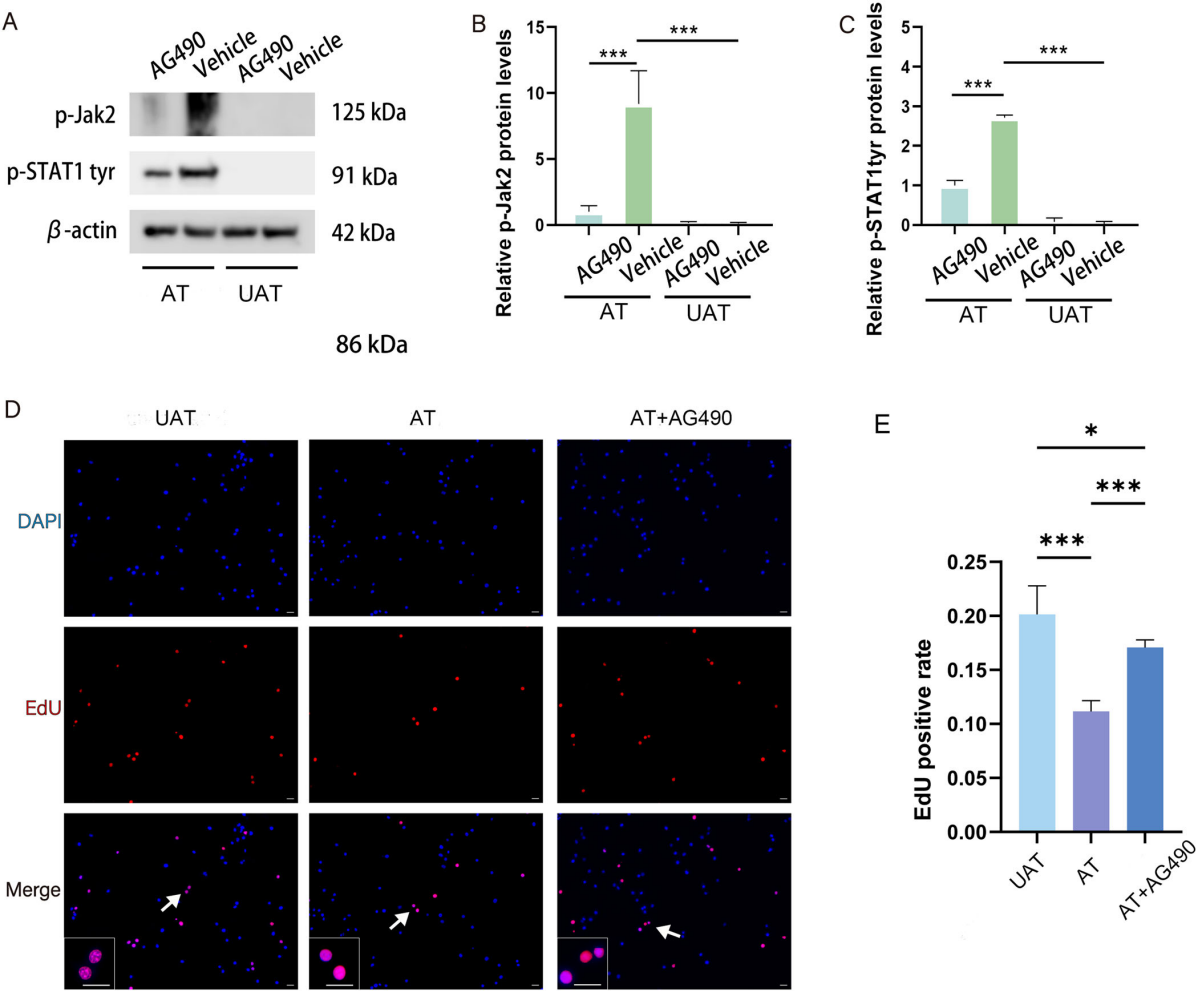
The JAK2/STAT1 pathway mediates the inhibitory effect of activated CD8 + T lymphocytes on NSC proliferation. (A) Representative western blot image of p‐Jak2‐tyr and P‐STAT1‐tyr expression in NSCs under different treatment conditions; (B and C) Relative protein expression of p‐Jak2‐tyr and P‐STAT1‐tyr (*n* = 3); (D) Representative immunofluorescence images of EdU+ NSCs under different treatment conditions. The image at the bottom left showed a magnification of the point of the arrow. Scale bar = 100 μm; (E) Statistical analysis of EdU+ NSCs under different treatment conditions (*n* = 5). AT, activated CD8 + T lymphocytes; AT + AG490, activated CD8 + T lymphocytes; AG490, a Jak2 inhibitor; UAT, unactivated CD8 + T lymphocytes. *p < 0.05, **p < 0.01, ***p < 0.001.

To further determine the role of the JAK2/STAT1 pathway in the inhibitory effect of activated CD8 + T lymphocytes on NSC proliferation, AG490, a JAK2 phosphorylation inhibitor, was added into the coculture medium. The results of Western blot analysis showed the levels of phosphorylated JAK2 and STAT1 at Tyr701 were significantly reversed when CD8 + T lymphocytes were activated (for p‐Jak2, AT + AG490 vs. AT+Vehicle, *p* < 0.001; for p‐STAT1‐tyr, AT + AG490 vs. AT+Vehicle, *p* < 0.001) (Figure 5A–C). Then, an EdU incorporation assay was performed to detect the proliferative ability of NSCs. The inhibitory effect of activated CD8 + T lymphocytes on NSC proliferation was also significantly reversed by AG490 (AT + AG490 vs. AT, *p* < 0.001) (Figure 5D,E).

## 4 Discussion

In this study, we demonstrated that activated peripheral CD8 + T lymphocytes can inhibit the proliferation of hippocampal NSCs in a time‐ and dose‐dependent manner. The inhibitory effect of CD8 + T lymphocytes on NSC proliferation may be achieved by secreting cytokines, especially IFN‐γ, as administering IFN‐γ neutralizing antibodies could attenuate this effect. Adding IFN‐γ directly to the coculture system also inhibited NSC proliferation, even without activating T cells. Additionally, the JAK2/STAT1 pathway in NSCs was activated by activated peripheral CD8 + T lymphocytes or exogenous IFN‐γ, and JAK2‐specific inhibitor can rescue the inhibitory effect of activated peripheral CD8 + T lymphocytes on NSC proliferation. These findings suggest that the activation of peripheral CD8 + T lymphocytes results in the secretion of inflammatory factors, especially IFN‐γ, which bind to IFNGR on NSCs, subsequently activate the JAK2/STAT1 pathway, and ultimately inhibit the proliferation of hippocampal NSCs.

In adult rodent and primate brains, two main areas contain NSCs and generate new neurons throughout adulthood: the SVZ of the lateral ventricles and the SGZ of the dentate gyrus (DG) in the hippocampus. Although they have the ability to perform neurogenesis, NSCs in different niche microenvironments generate different types of newborn neurons that perform different functions. NSCs in the SVZ area develop into olfactory nerves and participate in olfactory functions, whereas NSCs in the SGZ area develop into granulosa neurons in the DG and participate in cognitive and emotional functions. NSCs at different sites receive different regulatory signals, and the same regulatory signal may have different effects on NSCs at different sites. Manisha N Chandwani, et al reported that, in response to antiviral immunity, NSCs in the SGZ show greater proliferation and neurogenesis, whereas NSCs in the SVZ decline in both measures [18]. One study reported that IFN‐γ can inhibit the neurosphere formation of NSCs from the SVZ in vitro and decrease the number of newborn neurons in the olfactory bulb in vivo [19]. Another study showed that IFN‐γ promoted the differentiation of stem cells into neurons but could not compensate for its antiproliferative effect and ultimately inhibited neurogenesis in the SVZ [20]. In this study, we explored the direct role of CD8 + T cells on NSCs from SGZ.

It has been reported progressive decrease in the number of NSCs in the hippocampus with age [21]. A fraction of NSCs in the hippocampus proliferates more rapidly following activation and gets depleted thereafter [22], leading to exhaustion of this subpopulation early in life [23]. A further proportion is found to be long‐term self‐renewing NSCs, which can be preserved in the aged brain [24]. The NSC transcriptome sequencing results revealed that age‐related transcriptional changes are very mild. The main difference between these populations is that old NSCs tend to divide less frequently and remain quiescent to avoid complete exhaustion over time [25]. Once activated, however, young and old NSCs show similar proliferation and differentiation capacities. There is no difference in the morphology or function for newborn granular neurons derived from young and old NSCs [25]. CD8 + T‐cell‐mediated neuroinflammation can affect not only NSCs in adult or aged brains in the context of POCD but also younger or even newborn brains in cases such as encephalitis. Many studies have demonstrated that the number of NSCs in the hippocampi of aging mice decreases dramatically. To increase NSC extraction efficiency, the hippocampal tissues of neonatal mice were selected for the following research.

Different subsets of T cells may have different effects on NSCs during different pathophysiological processes. Th1 cells, a subtype of CD4 + T cells, are capable of inducing NPC cell death in a contact‐dependent manner in the pathogenesis of multiple sclerosis [26]. CD4 + T cells can also serve as negative modulators of neurogenesis after ischemic stroke [27]. In the embryonic cortex, CD8 + T cells, but not CD4 + T cells, inhibit the proliferation of NSCs [28]. However, CD4 + T cell‐derived IFN‐γ/IL‐10 promotes hippocampal neurogenesis in APP/PS1 and 3xTg‐AD mice [14]. The influenza vaccine recruits CD4 + T cells to the choroid plexus to promote hippocampal neurogenesis in pregnant mice [29], while CD8 + T cells infiltrating the brain, rather than CD4 + T cells, suppress neurogenesis following hepatitis B vaccination [30]. The same T cell subtypes at different sites also have different effects on NSCs. Spleen CD8 + T cells are essential for the effects of an enriched environment on promoting hippocampal neurogenesis [31]. Non‐CNS‐specific CD4 + T cells are prominently involved in the endogenous homeostatic principles that control baseline levels of hippocampal neurogenesis [32]. Our previous study revealed that CD8 + T cells could infiltrate the hippocampus and inhibit neurogenesis.

CD8 + T‐cells that infiltrate the brain parenchyma may be derived from the peripheral circulation or from the expansion of tissue‐resident memory T (Trm) cells. Trm cells are constantly retained in a given tissue with limited trafficking through the circulation, and the major function of Trm cells is immunosurveillance [33, 34]. CD8 + T‐cell infiltration into the brain starts at approximately 18 months of age in wild type mice [35]. Our previous study revealed that CD8 + T‐cell could infiltrate into hippocampus in 12‐month‐old mice that underwent major abdominal surgery but not that of the control mice [6]. A reasonable explanation for this phenomenon is that peripheral CD8 + T‐cells infiltrate the hippocampal parenchyma during pathological processes after surgery. Many studies also suggest that peripheral CD8 + T lymphocytes can cross the blood‒brain barrier or blood‒cerebrospinal fluid barrier, infiltrate certain brain tissues, and impair corresponding brain functions during several pathological processes, such as nerve injury [36], intracranial infection [37], stroke [38] and neurodegenerative disease [39]. Unlike Trm cells, once peripheral T lymphocytes enter the brain parenchyma and contact self or foreign antigens, they immediately develop an immune response and induce neuroinflammation [9, 40]. The model in our present study mimicked the immune response induced by CD8 + T lymphocytes infiltrating the hippocampus and was used to explore the effects of peripheral CD8 + T lymphocytes on NSCs.

Our previous studies showed that 12‐month‐old mice that underwent major abdominal surgery had T‐cell infiltration in the hippocampus, with an average density of 4 cells/section. With increasing age, the permeability of the blood‒brain barrier increases, and the levels of leukocyte chemokines in the brain parenchyma increase, which lead to a further increase in the number of lymphocyte infiltration into the brain during some pathological processes [36, 41]. Compared to thousands of hippocampal cells, the number of T cells infiltrating the hippocampus is small, as is the number of adult or elderly hippocampal NSCs. Gontier et al. reported that the density of NSCs expressing nestin in the hippocampus of 12‐month‐old mice was approximately 13 cells/section, and that of 18‐month‐old mice was 10 cells/section [42]. With increasing age, hippocampal nerve regeneration decreases. Some studies have also shown that the number of newborn neurons expressing doublecortin in the hippocampus of older mice ranges from 178 to 500 per DG [43, 44]. In some disease models, such as encephalitis and brain trauma, there are far more T cells infiltrating the brain′s parenchyma than NSCs. Taking the above factors into consideration, we chose a relatively reasonable ratio of T cells: NSCs to explore the effects of T lymphocytes on NSCs in aging mice in vitro.

Indeed, hippocampal neurogenesis involves a series of ongoing processes in which NSCs first give rise to transit‐expanding progenitors with transient amplification properties and subsequently differentiate into immature neurons, which further develop into mature dentate granule neurons [8]. New neurons are unlikely to have a unique impact on behavior at an early stage of development until they form mature functional connections with DG networks at 3–4 weeks post‐mitosis [45]. The results of our previous experiments revealed that CD8 + T lymphocytes infiltrated the hippocampus shortly after surgery but affected cognitive function 3–4 weeks after surgery [6]. Therefore, we speculated that CD8 + T lymphocytes infiltrating the hippocampus affect the early stages of hippocampal neurogenesis. The speculation that CD8 + T lymphocytes can inhibit NSC proliferation was subsequently validated by multiple experimental methods in this study. Consistent with our results, Ben W. Dulken et al. reported forcing CD8 + T lymphocytes entry into the brains of young mice by injecting a brain‐specific antigen was associated with a reduction in NSC proliferation in vivo [33]. Similar effects were observed when CD8 + T lymphocytes were cocultured with NSCs isolated from the cortices of mouse embryos or the SGZ of young mice [28, 33]. Wang et al reported that CD8 + T cells suppressed the proliferation of endogenous NSCs in the adult spinal cord and promoted the differentiation of NSCs into astrocytes, which promoted the formation of a glial scar [46].

The next question is how activated CD8 + T lymphocytes affect NSC proliferation. There are three possible ways: direct contact, indirect action through other cells, and indirect action through the secretion of cytokines. Although it has been reported that T cells could induced NSC death in a contact‐dependent manner [26], our previous study revealed that T lymphocytes infiltrating the brain parenchyma were dispersed and rarely aggregated in narrow neurogenic niches [6]. Therefore, we cocultured CD8 + T lymphocytes and NSCs in an indirect contact manner. One of the major effector functions of CD8 + T lymphocytes after contact with antigens is the secretion of IFN‐γ. As expected, the activated CD8 + T lymphocytes secreted more IFN‐γ. Many studies indicate that IFN‐γ can inhibit neurogenesis in the SVZ [19, 20], but the effect of IFN‐γ on neurogenesis in the SGZ is controversial. Zhang et al showed that the injection of IFN‐γ into the lateral ventricle can suppress NSC proliferation and stimulate the apoptosis of immature neurons in the SGZ [15]. This is consistent with our results that administering IFN‐γ neutralizing antibodies could attenuate the inhibitory effect of CD8 + T lymphocytes on NSC proliferation and adding IFN‐γ directly to the coculture system can also inhibit NSC proliferation, even without activating CD8 + T lymphocytes. However, another contradictory study showed that promoted T cell‐derived IFN‐γ via photobiomodulation therapy can improve neurogenesis in the SGZ [14]. The dose of IFN‐γ and exposure duration may be responsible for the different conclusion drawn. Distinct microenvironments in various pathological states could also be another possible reason. As IFN‐γ can act on various cells in the brain, such as NSCs and microglia, the final effect may be the result of a combined action [33].

Our results showed that IFNGR was highly expressed on the surface of NSCs in the hippocampus. The JAK‐STAT signaling pathway is a classical pathway involved in the signal transduction of IFNs and plays important roles in cell proliferation, differentiation, and apoptosis [47]. Distinct JAK‐STAT family members are activated by different cytokines, cell types and receptor types. Previous studies have shown that JAK1/JAK2 and STAT1/STAT3 are involved mainly in IFN‐γ signaling [48]. STAT1 regulates both the proliferation and differentiation fate of NSCs, while STAT3 seems to act only on differentiation fate of NSCs. The activation of STAT3 inhibits neuronal fate and promotes glial cell fate of NSCs [49, 50]. However, the reports regarding the effect of STAT1 activation on the differentiation fate of NSCs are inconsistent [20, 46]. In vitro studies have shown IFN‐γ can inhibit the proliferation of NSCs derived from spinal cord, embryo or SVZ by activating STAT1 [18, 20, 46]. However, it has been reported that IFN‐γ is unable to activate STAT3 in NSCs derived from SVZ, which is consistent with the finding in our study [20]. IFN‐γ can also affect the differentiation of NSCs by activating STAT1, but it is controversial whether IFN‐γ promotes neuronal fate or glial cell fate [20, 46]. Our study showed CD8 + T lymphocytes can activate JAK2 and STAT1 in NSCs by secreting IFN‐γ. After the addition of an inhibitor of JAK2, the inhibitory effect of CD8 + T lymphocytes on NSC proliferation was significantly attenuated.

This study has certain limitations. First, the main findings of this study were based on in vitro experiments, and further in vivo validations need to be carried out. Second, several key steps are needed for NSCs to develop into mature neurons, and here, we focused only on the effects of CD8 + T lymphocytes and IFN‐γ on the proliferative capacity of NSCs. Third, owing to the limitations of time and resources, no further in‐depth research on the effect of granzyme B was performed in this study. Finally, the microenvironment in vivo is complex, and we did not investigate the mechanism by which CD8 + T lymphocytes act indirectly on NSCs through other cells.

In summary, the present study revealed that activated peripheral CD8 + T lymphocytes can inhibit the proliferation of hippocampal NSCs by secreting IFN‐γ and subsequently activating the JAK/STAT signaling pathway in NSCs. The neuroinflammatory response and hippocampal neurogenesis have been shown to play an important role in many central nervous system disorders. Our study elucidates one of the many mechanisms that may be involved in the regulatory role of neuroinflammation in hippocampal neurogenesis and may provide novel ideas for the treatment of some central nervous system disorders.

## Author Contributions

**Xiaowei Li:** formal analysis, project administration, writing – original draft, writing – review and editing. **Xiaobin Sun:** project administration, writing – original draft. **Shiyu Hao:** data curation, methodology, project administration. **Guicheng Wang:** investigation, methodology. **Yanjing Guo:** data curation, formal analysis, methodology. **Yingxue He:** investigation, methodology. **Qidi Zhang:** project administration. **Zunsai Feng:** project administration. **Gongming Wang:** conceptualization, writing – review and editing. **Chengxiao Liu:** conceptualization, visualization, writing – review and editing.

## Ethics Statement

All experiments were approved by the Institutional Animal Care and Use Committee at Shandong Provincial Hospital, Shandong First Medical University (Jinan, China) (Ethical Approval Number: NSFC: No. 2021‐584).

## Conflicts of Interest

The authors declare no conflicts of interest.

## Supporting information


**Supplement Figure 1:** Activated CD8 + T cells inhibit NSC proliferation in a time‐dependent manner.

**Supplement Figure 2:** Granzyme B concentration in the medium after activation of T cells (*n* = 3).

## Data Availability

The data sets used and/or analyzed during the current study are available from the corresponding author on reasonable request.
