# Supplementary figures and images for: Activated Peripheral CD8 + T Lymphocytes Inhibit the Proliferation of Hippocampal Neural Stem Cells via the IFN‐γ/JAK/STAT Signaling Pathway
Source: Immun Inflamm Dis. 2025 Oct 28;13(10):e70287. doi: 10.1002/iid3.70287 (PMC12559668; doi:10.1002/iid3.70287)

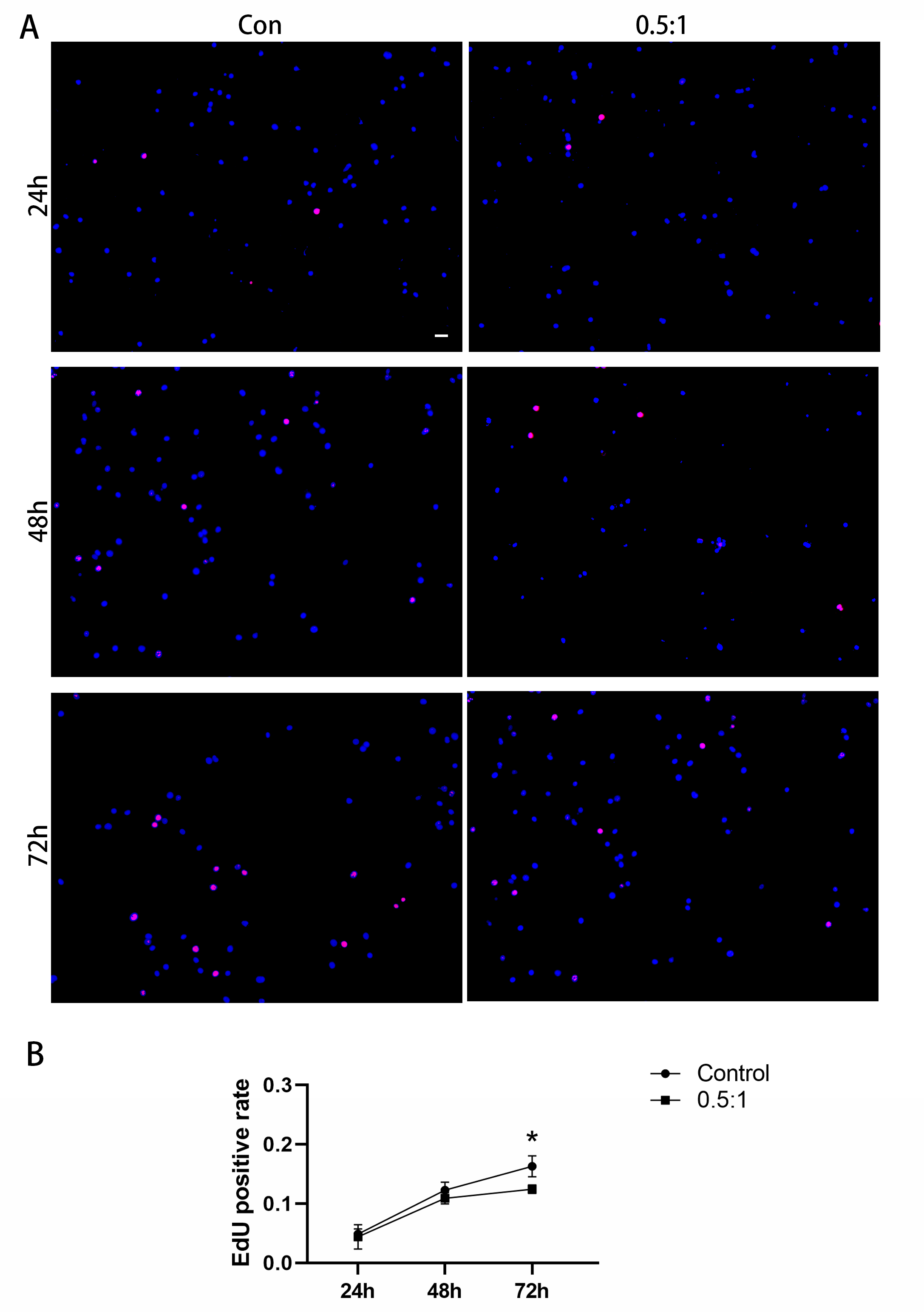

Supplement: Supplementary file 1 — Supplement Figure 1: Activated CD8 + T cells inhibit NSC proliferation in a time‐dependent manner. [file IID3-13-e70287-s002.tif]

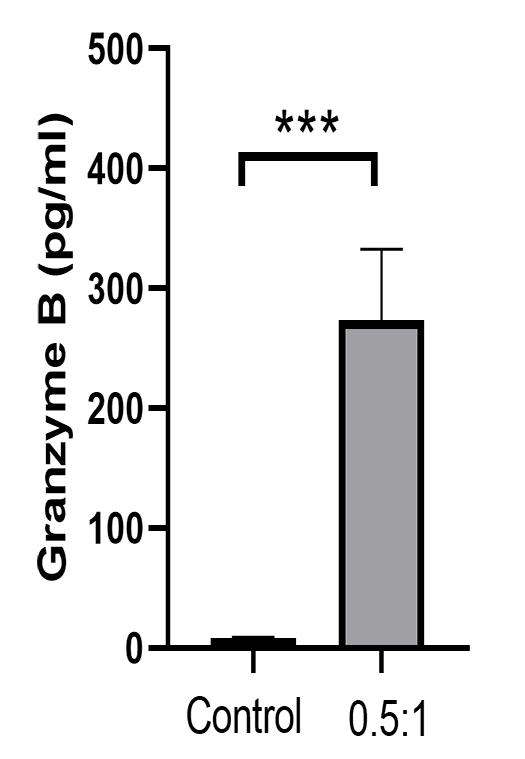

Supplement: Supplementary file 2 — Supplement Figure 2: Granzyme B concentration in the medium after activation of T cells (n = 3). [file IID3-13-e70287-s001.tif]
